# The COVID-19 Pandemic and International Trade: Temporary Turbulence or Paradigm Shift?

**DOI:** 10.1017/err.2020.29

**Published:** 2020-04-07

**Authors:** Lukasz GRUSZCZYNSKI

**Affiliations:** * Associate Professor, Kozminski University, Warsaw, Poland; Research Fellow, CSS Institute for Legal Studies, Budapest, Hungary; email: lgruszczynski@kozminski.edu.pl.

## I. Introduction

The COVID-19 epidemic has taken the world by surprise. Initially, it was seen as a Chinese, and later South-East Asian, problem. Decision-makers around the world apparently believed that the disease could be contained and controlled within the region, following a pattern that was evident in previous outbreaks, such as SARS. However, due to a combination of different factors of natural, political and regulatory character,^1^ the epidemic has quickly spread to other parts of the world and has been eventually recognised by the World Health Organization as a pandemic. The existing interconnectedness among countries obviously facilitated that expansion.

So far, the pandemic has been predominantly seen as a public health problem. As of 3 April 2020, there were more than 1 million confirmed cases globally, with almost 60,000 registered deaths.^2^ These numbers are expected to rise sharply in the near future. While some countries appear to be gaining control over the situation (eg China), others are either still battling to slow down the spread of the disease (eg Italy and Spain) or are in the early stages, which are typically characterised by exponential growth in cases (eg the USA and Poland). There are, however, other potentially equally serious consequences, the importance of which will only be appreciated as time passes. The current public health emergency will be followed by the mutually reinforcing economic and political crises that may ultimately lead to serious social disturbance as the costs of the pandemic will not only be high, but also unevenly distributed, both among countries and among different social groups within states.^3^

As far as the economic aspect is concerned, most experts expect to see in 2020 a global recession that may take a severe form for some countries or regions. For example, JP Morgan Research anticipates a two-quarter GDP contraction in the USA of between –10% and –25%, and for the Euro Area of between –15% and –22%.^4^ The pace and extent of potential recovery still remain open questions. The political crisis may manifest not only at the national level, by undermining the electoral support for current governments, but also at the regional or international level. In this context, some experts, for example, worry about the future of the European integration project, pointing to the inadequate response of the European institutions. Others see the COVID-19 pandemic as an existential threat to liberal democracy. Indeed, there are some early signs indicating that current autocratic tendencies may be strengthened in the future.^5^

International trade is also one of the potential victims of the current pandemic. As it is too early to assess the real impact of the various processes that are taking place now, the objective of this text is limited. Instead of identifying and analysing the probabilities of different scenarios, the intention is to highlight one possible course of action that seems to be emerging in the field. To this end, the two following sections discuss the short- and long-term consequences of the current pandemic for international trade. The final section offers some brief conclusions.

## II. Short-term consequences of the COVID-19 pandemic

The COVID-19 outbreak has already caused deep disruption to world trade, affecting both the supply and demand sides of the global economy. Many governments have ordered temporary closure of non-essential manufacturing facilities, while numerous corporations either have taken such measures voluntarily (eg because of the reduction in the supply of labour) or have simply decreased production due to disruptions in their supply chains. The impact of the COVID-19 pandemic is, however, most visible in the international service sector. The main victims are international tourism, passenger air travel and container shipping. Global financial transactions as well as information and communications technology services have also declined significantly.^6^ Moreover, according to the recent United Nations Conference on Trade and Development (UNCTAD) assessment, which is actually based on conservative assumptions, the COVID-19 outbreak will cause global foreign direct investments (service mode 3) to shrink by 5–15% in 2020.^7^ The demand side has also been affected as consumers around the globe are unwilling at the moment to spend their money. This phenomenon can be attributed to a common fear of loss of income (eg due to unemployment) and heightened uncertainty. Overall, one may expect to see a continued decline in the volume of international trade in the coming months. The extent of this decline is difficult to predict.

These past weeks have also seen a significant increase in states’ recourse to COVID-19-related trade policy measures. In particular, some countries have decided to establish export controls over certain medical products (eg medical ventilators, certain drugs, personal protective equipment) in the form of temporary export bans or the addition of licensing/authorisation requirements.^8^ Other countries, concerned with the security of their food supplies, have introduced export restrictions over specific agricultural products, and these decisions have generated genuine concerns about potential food shortages in the global market in the second part of the year.^9^ The problem appears sufficiently serious that it has led to a joint statement by the Directors-General of the Food and Agriculture Organization, the World Health Organization and the World Trade Organization (WTO), in which they noted that “uncertainty about food availability can spark a wave of [additional] export restrictions, creating a shortage on the global market”. In this context, they called on countries to ensure that their trade-related measures do not disrupt the food supply chain.^10^

However, it would be a mistake to think that the current epidemiological situation has only resulted in a wave of trade restrictions. The picture is much more complex. In fact, a number of states have recently removed or suspended some trade controls. For example, Argentina has suspended its anti-dumping duties on imports of certain medical products from China, while Canada has temporarily eliminated tariffs for specific categories of products if they are imported by public health agencies, hospitals and testing sites, or for use by first-response organisations.^11^ The aim of all of these measures is to ensure that there are sufficient supplies to domestic markets (either by decreasing exports or increasing imports). Interestingly, some trade restrictions have been reduced (at least temporarily) even between the USA and China, the two rivals that have been stuck in a trade war for the last two years. In particular, the USA has decided to exclude a range of medical protective gear and equipment from additional duties imposed previously under its Section 301, and new products may be added to that list in the future. Similarly, China has granted temporary exclusions for certain US goods (eg reagents or disinfectants) from its counter-duties.^12^

The pandemic has also slowed down the progress of various international trade initiatives around the globe, as states are currently preoccupied with the crisis. A good example is the new agreement between the USA, Mexico and Canada (so-called USMCA) that is supposed to replace the current NAFTA arrangement. Although it has already been ratified by all three parties, its entry into force depends on the successful implementation of its obligations at the national level. While the initial plan was slated for 1 June 2020, this initiation date is now unsustainable.^13^ Similar problems may be faced by the US–China Phase 1 trade deal concluded in January 2020 – the preliminary agreement that sets prerequisites for ending (again, at least temporarily) the trade war between the two countries. On its basis, China undertook to purchase more US goods and service, while the US agreed to lower some of its tariffs introduced for Chinese products between 2017 and 2019. It is unclear whether, in the present situation, China will be able to meet the required purchase thresholds, and equally whether the USA will be able to deliver a sufficient amount of goods and services.

On the other side of the Atlantic, talks between the UK and the European Union over future trade relations have also stalled.^14^ According to the withdrawal agreement, the transition period for the UK ends on 31 December 2020. If no deal is reached, the mutual trade relations will be governed by WTO rules. That seems to be a very unappealing option, particularly for the post-COVID-19 world, so one may expect to see (probably relatively soon) the extension of the deadline.

## III. Long-term consequences of the COVID-19 pandemic

The global economy is built on the specialisation of labour across countries. In line with the theory of comparative advantage, which provides the foundation for the current system of the international exchange of goods and services, such specialisation allows for maximisation of total output and improvement in welfare. The COVID-19 pandemic has shown, however, that clear benefits of the system come with costs. As noted by two commentators, “single-source providers, or regions of the world that specialize in one particular product, can create unexpected fragility in moments of crisis, causing supply chains to break down”.^15^ Such disruptions can have significant impacts, both on individual companies and on global systems of distribution. For example, China is a dominant global supplier of active pharmaceutical ingredients for many important medications. In 2018, it accounted for 95% of the US imports of ibuprofen, 91% of hydrocortisone, 40–45% of penicillin and 40% of heparin.^16^ Such a situation becomes particularly problematic in times of crisis when production facilities are not fully operational, while the demand of the domestic market may require countries to redirect part of their export. This is also true for other sectors, even if the consequences of possible disruptions are not so dramatic.

This newly discovered risk may eventually lead to profound changes in existing supply chains. The early signs of such a process have been visible over recent years with the Trump Administration pressuring American companies (albeit for different reasons) to move their production back to the USA, or at least to outside of China.^17^ These efforts have been only partially successful, but the current outbreak may trigger a more strenuous response. Interestingly, it seems that both private companies and governments may now be interested in introducing such modifications. From the point of view of private companies, shortening and diversifying supply chains can be a rational strategy that allows them to ensure smoother operations and eliminates the risk of supply shortages. For governments, this may be a way to limit dependence on one country (particularly in emergency situations) and as a consequence make them better prepared for future crises. This way of thinking is well illustrated by the recent statement from US Secretary of State Mike Pompeo during an interview in which he stressed the need to “fundamentally review our supply chains and make sure that we know those supply chains and have control over them for moments just like this”.^18^ It seems that this approach will be followed regardless of who wins the upcoming presidential election. In the past, regulatory initiatives aimed at reducing vulnerabilities in supply chains have attracted bipartisan support in the American Congress.^19^

Drawing on historical parallels, some commentators argue that the consequences of the pandemic will be even more far-reaching. They forecast a resulting deep and lasting transformation of the process of globalisation.^20^ The new world, as expected to emerge, will be characterised by tighter immigration rules, newly erected trade and investment barriers and technological decoupling, with a central role reserved for states rather than for international institutions (as it seems that only states are capable of offering solutions to existential challenges such as the COVID-19 pandemic).^21^ The probability of this scenario is further increased by various recent developments. It seems that some fundamental reorganisation of the global economy and international order has actually been going on for some time. In this context, it is worth noting that some multilateral institutions have already been marginalised. The WTO may serve here as a perfect example with its partially paralysed dispute settlement system.^22^ In response to the series of recent migration crises, immigration rules have also been strengthened in many countries. Global trade restrictions have been on the rise for last couple of years, and they are not limited to the economic relations between the USA and China.^23^ The European Union, which is traditionally very open to international trade, has recently taken a more assertive stance in its willingness to impose more vigorously its anti-dumping duties, countervailing measures and trade sanctions, as well as to undertake strategic investment screening.^24^ Technological decoupling – seen by both China and the USA in terms of competition for global technological supremacy – has been an important part of their trade war.^25^ A series of the recent competition proceedings by the European Commission against American technological companies also seems to constitute one of the elements of this process. Whether this will lead to the resurrection of national states (as suggested above) or rather to a segmentation of the world that will be based on regional economic blocs around local hegemons that compete against each other in the global power game is still an open question.^26^

## IV. Conclusions

Although some of the short-term consequences of the COVID-19 pandemic for international trade are serious, they do not appear to be unmanageable. From this perspective, one could expect that once the pandemic disappears (or is at least under control), international trade will go back to business as usual. However, in a different time frame, the potential impact of the pandemic may be more profound than initially anticipated, leading to structural changes in the process of economic globalisation. While the seeds of such a process were sown some time ago, the COVID-19 pandemic may exacerbate existing tendencies for states to turn inwards and compete more openly for economic and political dominance in the world. Whether this actually happens will greatly depend on the length and severity of the current pandemic. The bigger its impact, the greater are the chances that we will see the paradigm shift in international trade relations and governance.

## Footnotes

1 The size of the initial outbreak is due to the Chinese authorities ignoring, for political reasons, early signs of the unfolding epidemic, while many other countries have been late with their regulatory responses. COVID-19 as such has turned out to have a relatively high transmission rate, with a considerable number of infected people remaining symptomless, which facilitates new infections.

2 Cf John Hopkins University & Medicine, Coronavirus Resource Center <https://coronavirus.jhu.edu/map.html>.

3 For an interesting overview of different narratives on the COVID-19 pandemic and its consequences, see A Roberts and N Lamp, “Is the Virus Killing Globalization? There’s No One Answer”, *Barron’s*, 15 March 2020 <https://bit.ly/39EQiuB>.

4 JP Morgan, “Fallout from COVID-19: Global Recession, Zero Interest Rates and Emergency Policy Actions”, 27 March 2020 <https://www.jpmorgan.com/global/research/fallout-from-covid19>.

5 For example, the Hungarian Parliament has recently passed a law that gives virtually unlimited power to the Prime Minister (see L Gall, “Hungary’s Orban Uses Pandemic to Seize Unlimited Power”, *Human Rights Watch*, 23 March 2020 <https://bit.ly/2QYRIKg>). China is also using the current crisis to cement the power of the ruling communist party.

6 WTO, “Services Trade Barometer”, 11 March 2020 <https://bit.ly/39womJC> (note that those data cover January 2020 and the whole situation has worsened in the subsequent months as the epidemic unfolded).

7 UNCTAD, “Impact of the Coronavirus Outbreak on Global FDI”, March 2020 <https://bit.ly/2xH4bv2>.

8 In principle, those measures are WTO-compatible. While they may be regarded as prohibited quantitative restrictions on exports, they are potentially justified as necessary to protect public health. Of course, they need to be applied in a manner that does not discriminate between WTO Members and cannot constitute a disguised restriction on international trade.

9 S Nguyen, “Coronavirus: Vietnam Stockpiles Rice as Outbreak Spreads and Food Security Concerns Grow”, *South China Morning Post*, 28 March 2020 <https://bit.ly/2QZME89>.

10 “Joint Statement by QU Dongyu, Tedros Adhanom Ghebreyesus and Roberto Azevêdo, Directors-General of FAO, WHO and WTO”, 31 March 2020 <https://bit.ly/3433Uis>.

11 WTO, “COVID-19: Trade and Trade-Eelated Measures (as of 29 March 2020)” <https://www.wto.org/english/tratop_e/covid19_e/covid_measures_e.pdf>.

12 DW Layton, J Zhang and H Li, “The Impact of COVID-19 on the US–China Trade Relationship”, *Mayer Brown*, 13 March 2020 <https://bit.ly/39zCXE1>.

13 J McGregor, “Revised NAFTA Will Not Take Effect on June 1, as Trump Had Hoped”, *CBC*, 31 March 2020 <https://www.cbc.ca/news/politics/tuesday-nafta-june-coming-into-effect-1.5516490>.

14 S Payne, G Parker and J Brunsden, “Brexit Transition Deadline in Doubt as Talks Called Off”, *Financial Times*, 17 March 2020 <https://www.ft.com/content/14232572-686e-11ea-800d-da70cff6e4d3>.

15 H Farrell and A Newman, “Will the Coronavirus End Globalization as We Know It?”, *Foreign Affairs*, 16 March 2020 <https://fam.ag/2QYcdXg>.

16 D Palmer and F Bermingham, “U.S. Policymakers Worry about China ‘Weaponizing’ Drug Exports”, *Politico*, 20 December 2019 <https://politi.co/2QXHidx>.

17 JR Reed, “President Trump Ordered US Firms to Ditch China, but Many Already Have and More Are on the Way”, *CNBC*, 1 September 2019 <https://cnb.cx/3aLGh0p>. For an analysis of the American trade policy under Trump, see L Gruszczynski and J Lawrence, “Trump, International Trade and Populism” (2018) 49 Netherlands Yearbook of International Law 19.

18 “Secretary Michael R. Pompeo with Hugh Hewitt of the Hugh Hewitt Show”, US Department of State, 26 March 2020 <https://bit.ly/2UyjGyn>.

19 Cf J Whalen, “Commission That Advises Congress on China Warns of Prolonged Strategic Competition”, *The Washington Post*, 14 November 2019 <https://wapo.st/2UDfOMR>.

20 H James, “A Pandemic of Deglobalization?”, *Project Syndicate*, 28 February 2020 <https://bit.ly/3bK1o3e>.

21 I Bremmer, “Why COVID-19 May Be a Major Blow to Globalization”, *Time*, 5 March 2020 <https://time.com/5796707/coronavirus-global-economy>. For the contrary view, see Z Karabell, “Will the Coronavirus Bring the End of Globalization? Don’t Count on It”, *The Wall Street Journal*, 20 March 2020 <https://on.wsj.com/2WZW9rP>.

22 For an in-depth analysis of this crisis, see C Lo, J Nakagawa, T-F Chen (eds), *The Appellate Body of the WTO and Its Reform* (Amsterdam, Springer 2020).

23 Eg WTO, “Overview of Developments in the International Trading Environment. Annual Report by the Director-General (Mid-October 2018 to Mid-October 2019)”, 29 November 2019, WT/TPR/OV/22.

24 H von der Burchard, J Barigazzi and K Oroschakoff, “Here Comes European Protectionism”, *Politico*, 23 December 2019 <https://politi.co/39CruDH>.

25 C Ting-Fang and L Li, “The Great US–China Tech Decoupling: Where Are We Now?”, *Nikkei Asian Review*, 30 December 2019 <https://s.nikkei.com/2UAwCE2>.

26 See, eg, RD Kaplan, “Coronavirus Ushers in the Globalization We Were Afraid of”, *Bloomberg Opinion*, 20 March 2020 <https://bloom.bg/2USsBKa>.

